# Retrospective analysis on the immunopotentiating mechanism of an emulsion-based vaccine adjuvant on human antigen presenting cells

**DOI:** 10.3389/fimmu.2022.1086752

**Published:** 2023-01-09

**Authors:** Srinivasa Reddy Bonam, Peter Paul Platenburg, Jagadeesh Bayry

**Affiliations:** ^1^Institut National de la Santé et de la Recherche Médicale, Centre de Recherche desCordeliers, Sorbonne Université, Université de Paris, Paris, France; ^2^LiteVax B.V., Oss, Netherlands; ^3^Department of Biological Sciences & Engineering, Indian Institute of Technology Palakkad, Palakkad, India

**Keywords:** dendritic cells, adjuvant, cytokines, oil-in-water emulsion, CMS, human, CD4^+^ T cell, antigen presenting cell (APC)

## Abstract

We retrospectively analyzed the immunopotentiating mechanism of an oil-in-water (O/W) emulsion-based vaccine adjuvant LiteVax™ Adjuvant (LVA) that contains CMS (Maltose 4’-monosulphate 1,2,3,6,2’,3’,6’-heptadecanoic acid ester), squalane, Tween 80 in phosphate buffered saline. Despite being effective in animal models, the immunological mechanisms by which LVA exerts adjuvant function are not known. As dendritic cells (DC) are key for initiating and propagating the immune response, we have investigated the effect of LVA and of its components on the DC function. We show that CMS but not LVA significantly enhances the expression of DC activation-associated markers, cytokine secretion, and CD4^+^ T cell responses. On the other hand, CMS ZERO [non-sulphated sucrose fatty acid esters (ZERO)], used as a control, had no such activity. Our data identified the unique nature of CMS in LVA, and propose that LVA acts as a delivery system, and CMS acts as an immunostimulatory agent.

## Introduction

**Figure 1:**
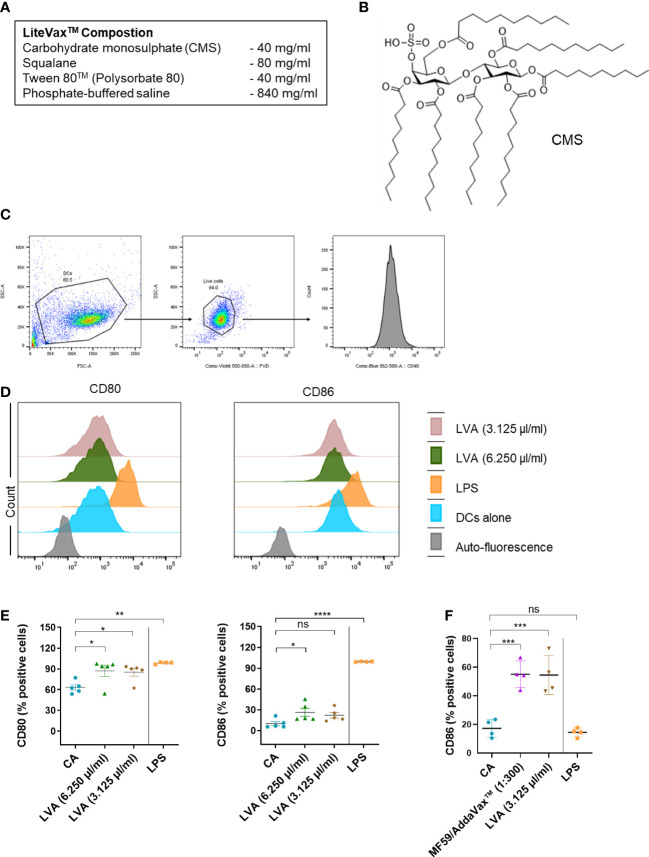
Immunological mechanism of action of LiteVax™ (LVA) adjuvant on antigen presenting cells. **(A)** Composition of LVA. **(B)** Structure of active constituent of LVA, CMS: Maltose 4’–monosulphate 1,2,3,6,2’,3’,6’–heptadecanoic acid ester. **(C–E)** Effect of LVA on dendritic cell maturation. Monocytes were isolated from human peripheral blood mononuclear cells. Monocytes were cultured with granulocyte–macrophage colony–stimulating factor (GM–CSF, 1000 IU/10^6^ cells) and IL–4 (500 IU/10^6^ cells) in RPMI 1640 complete medium (10% fetal calf serum and 1% penicillin–streptomycin) for 5 days to obtain immature dendritic cells. Immature dendritic cells (0.5x10^6^ cells/ml) were seeded in the 24–well plate and left untreated (CA) or treated with LPS (100 ng/ml, a positive control), LVA (3.125 µl/ml, which contains 125 µg of CMS, or 6.250 µl/ml, which contains 250 µg of CMS) for 48 h. Gating strategy is presented **(C)**. Representative histograms **(D)** with scatter plots **(E)** of data presented as mean ± SEM (n=4–5) values of expression (% positive cells) of CD80 and CD86. **(F)** Effect of LVA on monocytes activation. Monocytes were cultured in RPMI complete medium without GM–CSF/IL–4 and were either not treated or treated with LPS (100 ng/ml), AddaVax™/MF59 (1:300 v/v, a squalene–based oil–in–water nano–emulsion as a control), LVA (3.125 µl/ml) for 48 h. After incubation, cells were subjected to phenotyping by flow cytometry and the expression of CD86 (% positive cells) was presented as mean ± SEM (n=4 donors). Statistical significance as determined by one–way ANOVA with Dunnett’s multiple comparisons post–test. *P < 0.05, **P < 0.01, ***P < 0.001, ****P < 0.0001, ns, not significant. CA, cells alone, LPS, lipopolysaccharide.

Adjuvants have been considered as essential components of several type of vaccines, which enhance and potentiate the intensity, duration and sometimes type of immune response (1–3). LiteVax™ Adjuvant (LVA) is an oil-in-water (O/W) emulsion (**Figure 1A**), and contains CMS (Maltose 4’-monosulphate 1,2,3,6,2’,3’,6’-heptadecanoic acid ester) (**Figure 1B**), squalane, Tween 80 in PBS. LVA contains the third generation of Synthetic carbohydrate fatty acid sulphate esters (CFASE). CoVaccine HT™ adjuvant is the precursor of LVA (4–6). Both adjuvants are similar types of formulations (i.e., sub-micron emulsion of squalane-in-water) and contain the same components, i.e. CFASE, squalane and Tween 80. CFASE is a mixture of non-sulphated sucrose fatty acid esters (ZERO), sucrose fatty acid monosulphate esters (CMS or MONO) and sucrose fatty acid polysulphate esters (POLY). The principle difference between CoVaccine HT™ and LVA is the different concentrations of the highly reactogenic POLY. CoVaccine HT™ contains >30% POLY whereas LVA contains <1% POLY in the formulation. It has been shown that POLY is 100- to 1000-fold more reactogenic than MONO (CMS), while the adjuvant activity is similarly high (4). A strong synergy between CFASE and squalane-in-water emulsion has been shown (5) and strong adjuvant activity of LVA suggests synergistic cooperation between CMS and the squalane emulsion. CMS is non-reactogenic (in rabbits), non-haemolytic, chemically and physically stable, either alone or with formulations, and is highly compatible with emulsion-based delivery systems. It has proved its effectiveness as an adjuvant against influenza (inactivated whole-virion H7N9 in ferrets), and malaria (R0.10C; *Plasmodium falciparum* gametocyte extract) (4). LVA is currently in the pipeline for entering into the clinical trial. However, the immunological mechanisms by which LVA exerts adjuvant function are not known. As dendritic cells play a key role in initiating and propagating the adjuvant-mediated immune response by virtue of being sentinels of the immune system and as professional antigen-presenting cells (7–9), we have investigated the effect of LVA and of its components on the dendritic cell function. Our data identified the unique nature of CMS in LVA, and propose that LVA emulsion acts as a delivery system and CMS acts as an immunostimulatory agent.

## Results

### LVA induces co–stimulatory molecules in human dendritic cells and monocytes

We first performed dose–response analyses of LVA on the purified monocyte–derived human dendritic cells to decipher its cytotoxicity. Dendritic cells were differentiated from the monocytes of healthy donors in the presence of granulocyte macrophage colony stimulatory factor and IL–4 as previously described (10). Buffy coats of healthy donors were used for the experiments with relevant ethical permission (EFS–INSERM ethical committee permissions 18/EFS/033). The results revealed that at higher concentrations, LVA is cytotoxic to immature human dendritic cells (**Supplementary Figure 1A**). However, we noted that the cytotoxicity of the LVA at higher doses is related to the presence of a higher concentrations of Tween 80™ rather than CMS (**Supplementary Figure 1B**) (11). The data also suggested that LVA that contains CMS concentration less than 500 µg/ml (12.5 µl of LVA) is ideal for the mechanistic immunological evaluation. Therefore, we treated the human immature monocyte–derived dendritic cells with LVA containing 250 µg/ml (6.250 µl of LVA) or 125 µg/ml (3.125 µl of LVA) of CMS, and found that LVA could marginally enhance the B7 co–stimulatory molecules CD80 and CD86 on the dendritic cells (**Figures 1C–E**). Though the other activation markers tested (HLA–DR, CD40, CD54 and CD83) did not show any significant changes (**Supplementary Figure 2A**), the cytokine analysis also revealed a stimulatory effect of LVA on dendritic cells, i.e., an increased secretion of pro–inflammatory cytokine IL–6 and decreased expression of anti–inflammatory cytokine IL–10 (**Supplementary Figure 2B**). Furthermore, enhanced expression of co–stimulatory molecule CD86 was also observed when LVA was added to monocytes (**Figure 1F**). These data confirmed the partial activation of human dendritic cells by LVA.

### CMS is responsible for the immunostimulatory activity of LVA on the dendritic cells

**Figure 2:**
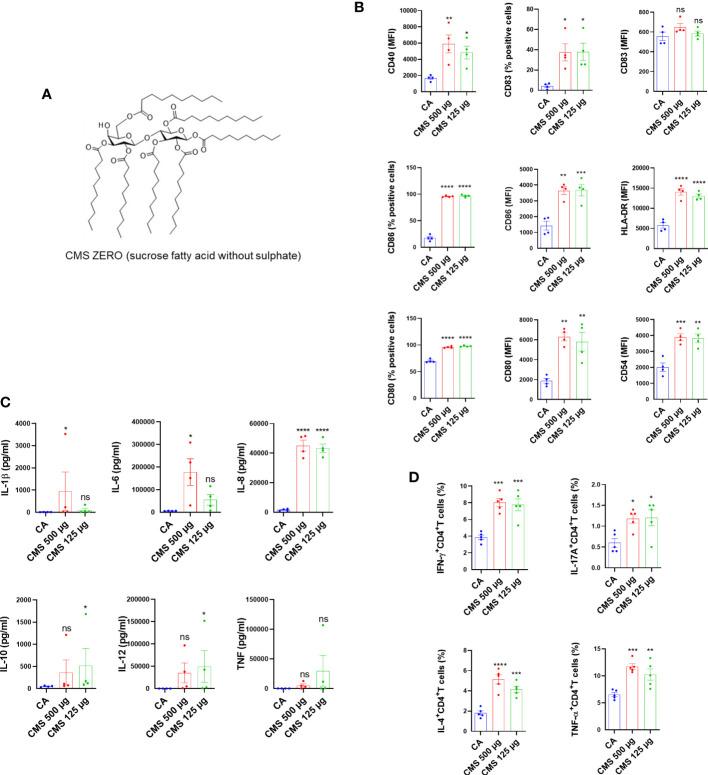
Identification of the component responsible for the immunostimulatory activity LiteVax™ (LVA) adjuvant on human dendritic cells. **(A)** CMS Zero (sucrose fatty acid without sulphate). **(B)** Effect of CMS on the expression of surface markers of human dendritic cells. Monocyte–derived dendritic cells (0.5x10^6^ cells were cultured with GM–CSF/IL–4 and were treated with either CMS 500 µg/ml, CMS 125 µg/ml for 48 h. The phenotype of dendritic cells was analyzed by flow cytometry. Data present values of expression (% positive cells or median fluorescence intensities, MFI) of CD40, CD54, CD80, CD83, CD86 and HLA–DR. Data are from n=4 donors with two independent experiments and are presented as mean ± SEM. **(C)** Secretion of cytokines (mean ± SEM, n=4) of IL–1β, IL–6, IL–8, IL–10, IL–12p70, and TNF–α (all in pg/ml) by treated dendritic cells. **(D)** Effect of CMS on the dendritic cell–mediated CD4^+^ T cell responses. Dendritic cells were cultured with GM–CSF/IL–4 and were treated CMS 500 µg/ml or CMS 125 µg/ml for 48 h. Dendritic cells were washed and co–cultured with purified allogeneic CD4^+^ T cells (1:10 ratio) for six days. After six days, cells were subjected to staining for the intracellular cytokines for Th1 (IFN–γ^+^CD4^+^), Th17 (IL–17A^+^CD4^+^), Th2 (IL–4^+^CD4^+^) cells, and TNF–α–secreting CD4^+^ T cells (TNF–α^+^CD4^+^). Data were presented as mean ± SEM (n=5 independent donors with three independent experiments). Statistical significance as determined by one–way ANOVA with Dunnett’s multiple comparisons post–test. *P < 0.05, **P < 0.01, ***P < 0.001, ****P < 0.0001, ns, not significant. CA, cells alone.

Further, we investigated the effect of individual components of LVA (CMS or MONO, ZERO (**Figure 2A**), solvent control [3.125 µl of 0.4% DMSO in phosphate buffered saline and 2% Tween 20, used to dissolve the CMS]) on the dendritic cells. The results revealed that CMS at 500 µg/ml and 125 µg/ml concentrations significantly enhanced the expression of co–stimulatory molecules (CD80 and CD86), antigen–presenting molecule (HLA–DR), adhesion marker (CD54), and marker associated with the terminal maturation of dendritic cells (CD83) (**Figure 2B**). However, either CMS ZERO or solvent control did not induce dendritic cell maturation markers (**Supplementary Figures 3A, B**) We observed that both the concentrations of CMS (either 500 µg/ml or 125 µg/ml) were equivalent in their ability to stimulate dendritic cells. Similarly, an increased secretion of cytokines (IL–6 and IL–8) was observed from the CMS–activated dendritic cells (**Figure 2C**). However, the induction of other cytokines like IL–10, IL–1β and IL–12 was not consistent and was observed with either of the concentrations of CMS (**Figure 2C**).

### CMS–stimulated dendritic cells enhance effector CD4^+^ T cell responses

Further, to validate that induction of maturation markers on dendritic cells by CMS is associated with an enhanced T cell stimulatory ability, CMS–treated dendritic cells were washed and cultured with allogenic CD4^+^ T cells for six days (1:10 = dendritic cell: CD4^+^ T cells). The intracellular cytokine analyses revealed that CMS–stimulated dendritic cells enhanced CD4^+^ T cell effector responses as evidenced by the increased frequencies of IFN–γ^+^CD4^+^ T, IL–4^+^CD4^+^ T, IL–17^+^CD4^+^ T, and TNF–α^+^CD4^+^ T cells (**Figure 2D**). Consistent with the dendritic cell data, CMS at both the concentrations induced similar CD4^+^ T cell responses.

## Discussion

Glycolipid–based adjuvants, such as QS–21 (from *Quillaja Saponaria)* and Monophosphoryl–Lipid A have been widely used in human vaccines for infectious diseases and cancer (1, 12–14). Despite the induction of potent immune responses, including both cell–mediated and humoral immunity, mild toxicity is a limiting factor for many glycolipid–based adjuvants (2, 15). The emergence of novel viruses and their variants cautions the scientific community for the preparedness with inclusive vaccine adjuvants (16). Safe and potent glycolipid–based adjuvants could play a key role in the development of safe and effective vaccines.

This study aims to understand the mechanisms underlying the efficacy of LVA adjuvant. Despite being effective against influenza and malaria (4), there is no known mechanism of action on human immune cells. We demonstrated that LVA induces co–stimulatory signals in human dendritic cells and monocytes, a crucial immune stimulatory induction of co-stimulatory molecules. Nonetheless, we wanted to investigate the component responsible for the induction of co-stimulatory molecules in question. CMS is an essential component of the LVA adjuvant, as demonstrated by studies of its individual constituents. These data indicate that the *in vivo* efficacy of LVA (published elsewhere) (4) was due to the active component of CMS, and under *in vitro* conditions LVA likely masks the CMS interaction with the dendritic cells. To investigate the role of the monosulphate group, we treated immature dendritic cells with CMS ZERO (non–sulphated sucrose fatty acid ester) and discovered that a change in the structure of CMS eliminated its immunostimulatory potential in terms of dendritic cell maturation and dendritic cell–mediated CD4^+^ T cell responses (**Supplementary Figures 3A, B**), thereby excluding a structural–activity relationship of CMS. Additionally, neither maturation of dendritic cells nor CD4^+^ T cell responses were observed in the solvent control used to dissolve the CMS (**Supplementary Figures 3A, B**).

Although a number of strategic vaccine adjuvants are capable of eliciting an immune response, their distinguishing characteristics are limited to specific antigens relative to other pathogen variants. To increase their efficacy, the antigens must be supplemented with vaccine adjuvants that are inclusive. We confirmed that CMS is an immunostimulant component of the LVA adjuvant. Our findings suggest that the *in vivo* immunopotentiating properties of LVA are due to the effect of CMS on antigen–presenting cells and subsequent T cell responses. The CMS molecules in LVA are transported by the squalane droplets. Known adjuvants such as MF59, ASO3, SE, and GLA–SE contain squalene emulsions or squalene–in–water without CMS, and *in vivo*, a synergistic collaboration between squalene and CMS precursors has been demonstrated (16, 17). The molecular mechanisms by which LVA and CMS act as a delivery system and immunostimulant, respectively, require further investigation. The aforementioned findings should also aid in the design and development of novel, effective vaccine formulations.

## Methods

### Ethics

The protocol (18/EFS/041) was approved by the ethical committee EFS–INSERM Paris.

### Monocytes isolation and LVA treatment

Buffy coats of healthy donors (age ranging from 30 to 50 years) were purchased from Centre Trinité, L'Établissement Français du Sang, Paris. Peripheral blood mononuclear cells (PBMCs) were isolated from the buffy coats by using a Ficoll density gradient centrifugation. Monocytes were isolated from PBMCs by using CD14 MicroBeads *via* magnetic cell separation with MACS system (Miltenyi Biotec). 0.5x10^6^ cells/ml monocytes were seeded in the 24–well plate followed by left untreated or treated with AddaVax™ (1:300 v/v and 12.5 µl/ml [concentration of squalene in AddaVax™ that is equal to the concentration of squalane in LVA]), LPS (100 ng/ml), LVA (125 µg CMS, 3.125 µl/ml) for 48 h. After incubation, monocytes were processed for surface staining of the molecules.

### Generation and culture of dendritic cells

Monocytes were isolated as previously described, and were cultured with granulocyte–macrophage colony stimulatory factor (GM–CSF, 1000 IU/10^6^ cells) (Miltenyi Biotec) and IL–4 (500 IU/10^6^ cells) (Miltenyi Biotec) in RPMI–1640 supplemented with 10% fetal calf serum (FCS), 1% penicillin–streptomycin for 5 days to obtain immature dendritic cells. The obtained immature dendritic cells (0.5×10^6^ cells/mL) were cultured with GM–CSF and IL–4 and were either unstimulated (cells alone, CA) or stimulated with LPS (100 ng/ml, *E. coli* 055:B5, Sigma–Aldrich), LVA (6.250 µl/ml or 3.125 µl/ml, which contains 250 µg or 125 µg of CMS, respectively) for 48 h. In some experiments, immature dendritic cells were treated with CMS at 500 µg/ml and 125 µg/ml for 48 h. After the incubation period, cell–free supernatants were stored for the various cytokine analyses and cells were processed for surface staining of various markers.

### Flow–cytometry

The following antibodies were used for the flow cytometry. CD80–PE, clone: L307.4, BD Biosciences, CD86–FITC, clone: FUN–1, BD Biosciences, CD40–PE, clone: MAB89, Beckman Coulter, CD54–APC, clone: HA58, BD Biosciences, CD83–APC, clone: HB15e, BD Biosciences, and HLA–DR–APC, clone: G46–6, BD Biosciences. The cells were acquired using flow cytometer (LSR II, BD Biosciences). BD FACS DIVA and FlowJo were used to analyze the expression of various molecules.

### Co–culture of dendritic cells and CD4+ T cells

Following the treatment of dendritic cells as described above, they were subjected to a mixed lymphocyte reaction with allogenic CD4^+^ T cells at a ratio of 1:10 (dendritic cells to T cell) for 5 days in serum–free X–VIVO medium. The CD4^+^ T cell isolation kit (from Miltenyi Biotec) was used to isolate allogenic CD4^+^ T cells from PBMCs. After six days of co–culture, cells were washed and stimulated for 4 hours with phorbol 12–myristate 13–acetate (50 ng/ml/0.5x10^6^ cells) and Ionomycin (500 ng/ml/0.5x10^6^ cells) (Sigma–Aldrich), as well as GolgiStop (BD Biosciences). Cells were stained for surface molecule (CD4) followed by fixation and permeabilization using Fixation/Permeabilization kit (eBioscience) and intracellular staining (IFN–γ, IL–4, IL–17A, and TNF–α). The following antibodies were used for the staining. IFN–γ–FITC, clone: 4S.B3, BD Biosciences, CD4–PerCP, clone: SK3, BioLegend, IL–17A–PE, clone: eBio64cap17, eBiosciences, IL–4–PE, clone: MP4–25D2, BD Biosciences, and TNF–α–APC, clone: cA2, Miltenyi Biotec.

### ELISA

Cell–free culture supernatants were analyzed for the cytokines IL–1β, IL–6, IL–8, IL–10, IL–12, and TNF–α (ELISA Ready–SET–Go, eBioscience).

### Statistical analysis

As described in the figure legends, the studies were conducted using cells from multiple independent donors. Data among the multiple groups were analyzed by one–way analysis of variance (ANOVA) with Dunnett’s multiple comparison test by using Prism 8 (GraphPad Software Inc, CA).

## Data availability statement

The original contributions presented in the study are included in the article/**Supplementary Material**. Further inquiries can be directed to the corresponding author.

## Ethics statement

The studies involving human participants were reviewed and approved (18/EFS/041) by the ethical committee EFS–INSERM, Paris. Written informed consent for participation was not required for this study in accordance with the national legislation and the institutional requirements.

## Author contributions

SB performed the experiments. SB and JB analyzed the data. PP provided essential tools. SB drafted the first version of the paper. All authors contributed to the article and approved the submitted version.
