# Supplementary material for: Retrospective analysis on the immunopotentiating mechanism of an emulsion-based vaccine adjuvant on human antigen presenting cells
Source: Front Immunol. 2023 Jan 9;13:1086752. doi: 10.3389/fimmu.2022.1086752 (PMC9868768; doi:10.3389/fimmu.2022.1086752)

Supplementary Material

**Retrospective analysis on the immunopotentiating mechanism of an emulsion-based vaccine adjuvant on human antigen presenting cells**

Srinivasa Reddy Bonam^1^, Peter Paul Platenburg^2^, Jagadeesh Bayry*^,1,3^

^1^Institut National de la Santé et de la Recherche Médicale, Centre de Recherche des

Cordeliers, Sorbonne Université, Université de Paris, Paris, France

^2^LiteVax B.V., Oss, The Netherlands

^3^Department of Biological Sciences & Engineering, Indian Institute of Technology Palakkad, Palakkad, India

*Author for correspondence: [jagadeesh.bayry@crc.jussieu.fr](mailto:jagadeesh.bayry@crc.jussieu.fr), [bayry@iitpkd.ac.in](mailto:bayry@iitpkd.ac.in)

**Figure S1. Cytotoxic effects of LiteVax^TM^ (LVA) adjuvant on dendritic cells and monocytes.** (**A)** Immature dendritic cells (DC, 0.5x10^6^ cells/ml) were seeded in the 24-well plate and left untreated (CA) or treated with LPS (100 ng/ml), LVA (from 50 µl/ml to 3.125 µl/ml, which contains 2000 µg/ml to 125 µg/ml of CMS) for 48 h. Dendritic cell viability was measured by using fixed viable dye. (**B)** Monocytes were either non-treated or treated with LPS (100 ng/ml), AddaVax™/MF59 (1:300 v/v, a squalene-based oil-in-water nano-emulsion as a control), LVA (3.125 µl/ml, which contains 125 µg/ml of CMS), Squalane (250 µg/ml), and CMS (125 µg/ml) + Tween 80 (250 µg/ml) for 48 h. The cells were stained with fixed viable dye and gated on monocytes for measuring the viability.


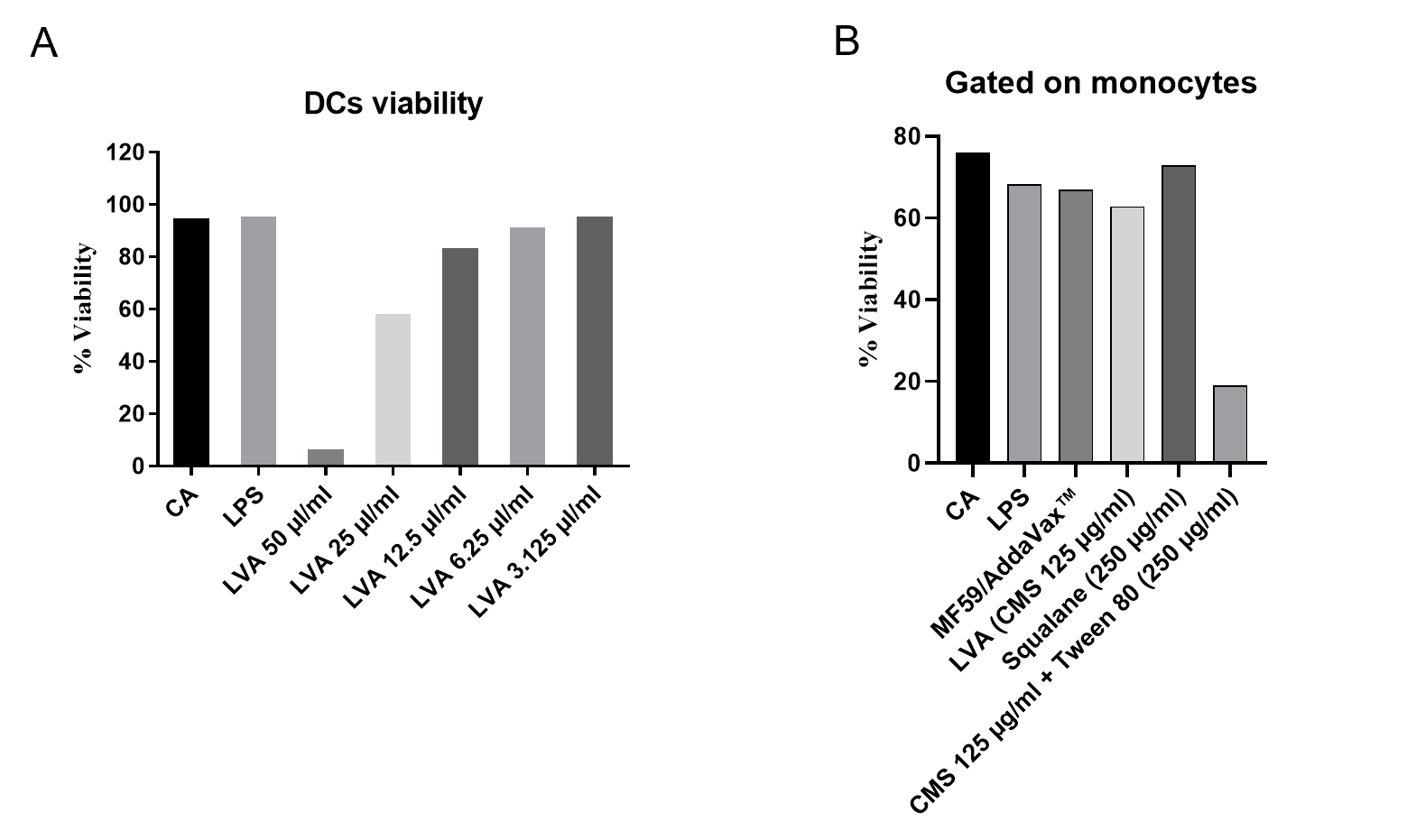


**Figure S2. Effect of LVA on dendritic cell maturation and cytokine secretion.** (**A)** Immature monocyte-derived dendritic cells (0.5x10^6^ cells/ml) were cultured in RPMI 1640 complete medium (10% fetal calf serum and 1% penicillin-streptomycin) with GM-CSF and IL-4 and were either unstimulated (cells alone; CA) or stimulated with LPS (100 ng/ml), LVA (3.125 µl/ml, which contains 125 µg of CMS or 6.250 µl/ml, which contains 250 µg of CMS) for 48 h. The expression of HLA-DR, CD40, CD54 (median fluorescence intensities, MFI) and CD83 (% positive cells) was analyzed. Data were presented as mean ± SEM and were from 4-5 independent donors **(B)** Secretion (mean ± SEM, n=4) of IL-6 and IL-10 by treated dendritic cells. Statistical significance as determined by one-way ANOVA with Dunnett's multiple comparisons post-test. **P* < 0.05; ***P* < 0.01; ****P* < 0.001; *****P* < 0.0001; ns, not significant. Abbreviation: CA, cells alone; LPS, lipopolysaccharide.


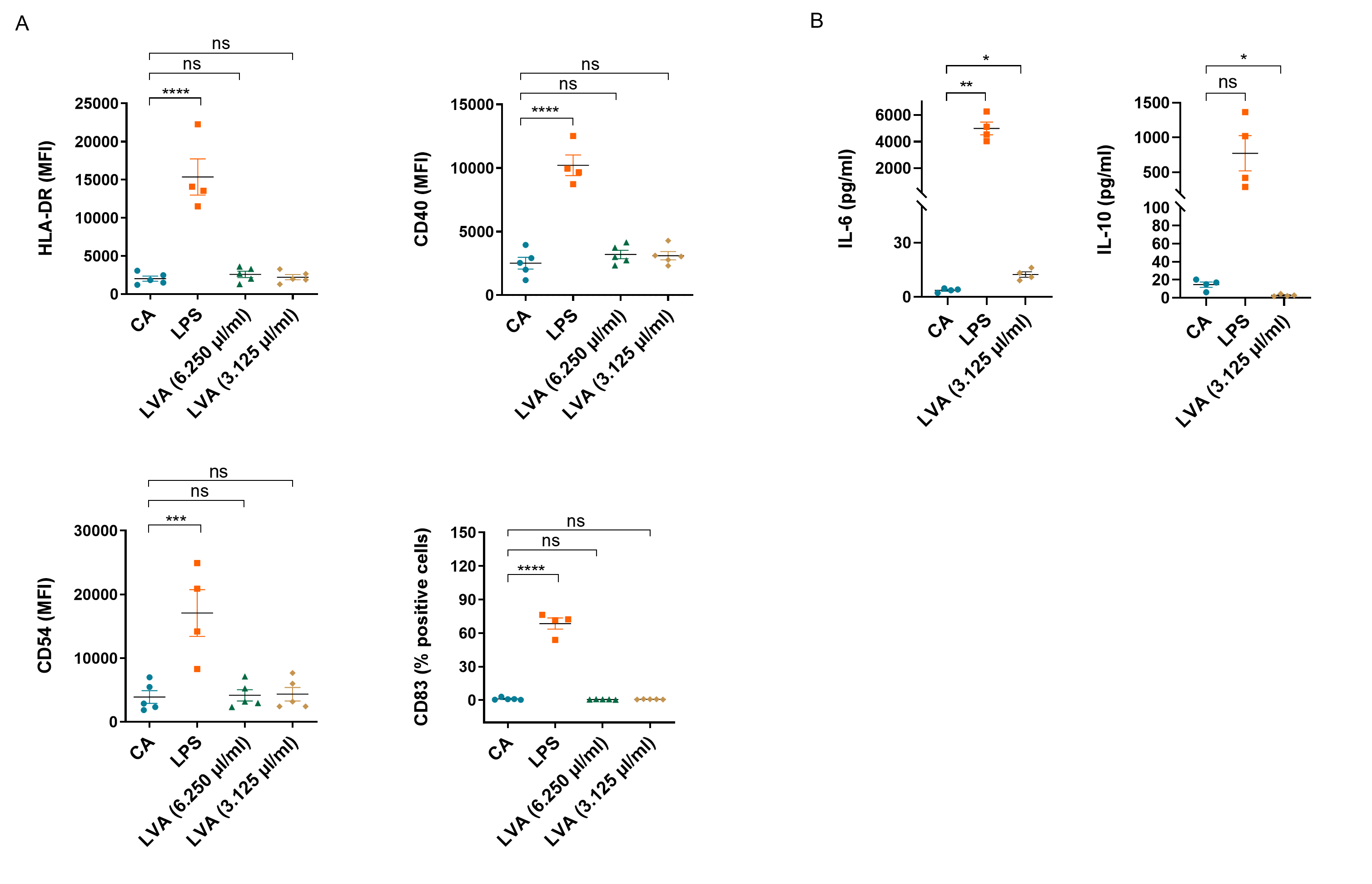


**Figure S3. Immunostimulatory effect of CMS ZERO and solvent control on human dendritic cells and CD4^+^ T cell responses**. (**A)** Effect of CMS ZERO and solvent control (Sol ctrl; 0.4% DMSO in phosphate buffered saline and 2% Tween 20) on the expression of surface markers of human dendritic cells. Monocyte-derived dendritic cells (0.5x10^6^ cells) were cultured with GM-CSF/IL-4 and were either unstimulated (cells alone; CA) or treated with either CMS ZERO 500 µg/ml or 125 µg/ml, Sol ctrl (3.125 µl) for 48 h. The phenotype of dendritic cells was analyzed by flow cytometry. Data (mean ± SEM) present values of expression (median fluorescence intensities, MFI) of CD40, CD54, CD80, CD83, CD86 and HLA-DR. Data were from n=4-6 donors with two independent experiments. The values are not statistically significant as determined by one-way ANOVA with Dunnett's multiple comparisons post-test. (**B)** Effect of CMS ZERO and Sol ctrl on the dendritic cell-mediated CD4^+^ T cell responses. Dendritic cells were cultured with GM-CSF/IL-4 and were treated CMS ZERO (500 µg/ml or 125 µg/ml) or Sol ctrl (3.125 µl) for 48 h. Dendritic cells were washed and co-cultured with purified allogeneic CD4^+^ T cells (1:10 ratio) for six days. After six days, cells were subjected to staining for the intracellular cytokines for Th1 (IFN-γ^+^CD4^+^), Th17 (IL-17A^+^CD4^+^), Th2 (IL-4^+^CD4^+^) cells, and TNF-α-secreting CD4^+^ T cells (TNF-α^+^CD4^+^). Data (mean ± SEM) were from 5-6 donors with three independent experiments. The values are not statistically significant.


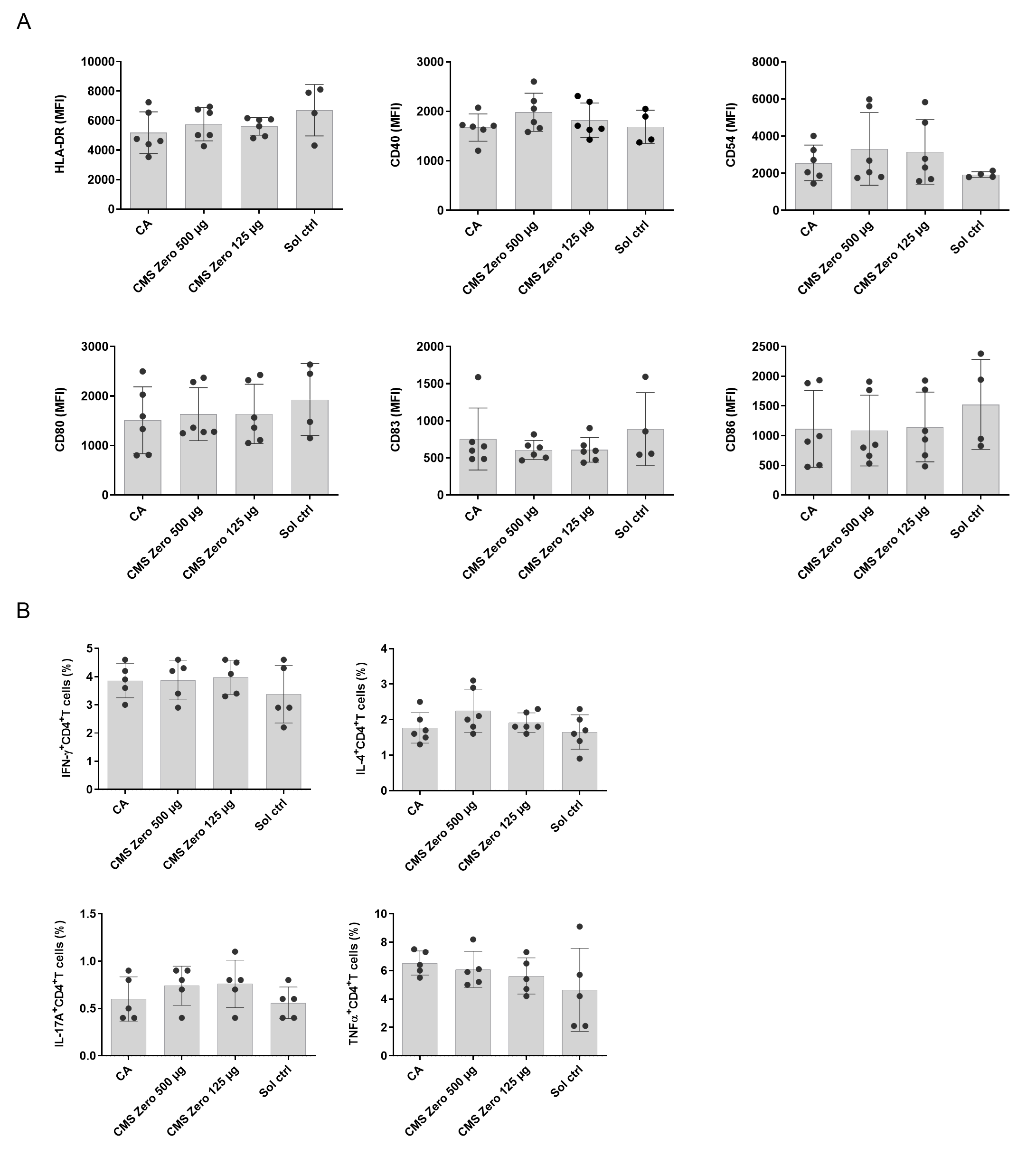

Supplement: Supplementary file 1 [file DataSheet_1.docx]
